# Grapefruit Extract-Mediated Fabrication of Photosensitive Aluminum Oxide Nanoparticle and Their Antioxidant and Anti-Inflammatory Potential

**DOI:** 10.3390/nano12111885

**Published:** 2022-05-31

**Authors:** Kholoud A. Bokhary, Farah Maqsood, Musarat Amina, Amal Aldarwesh, Hanan K. Mofty, Hanan M. Al-yousef

**Affiliations:** 1 Department of Optometry and Vision Science, College of Applied Medical Science, King Saud University, Riyadh 11451, Saudi Arabia; kbokhary@ksu.edu.sa (K.A.B.); fmaqsood@ksu.edu.sa (F.M.); aaldarweesh@ksu.edu.sa (A.A.); hmofty@ksu.edu.sa (H.K.M.); 2 Department of Pharmacognosy, College of Pharmacy, King Saud University, Riyadh 11451, Saudi Arabia; halyousef@ksu.edu.sa

**Keywords:** grapefruit peel, aluminum oxide nanoparticle, green synthesis, antioxidant, anti-inflammatory, immunomodulatory

## Abstract

Aluminum oxide nanoparticles (Al_2_O_3_ NPs) were synthesized using a simple, eco-friendly green synthesis approach in an alkaline medium from the extract of grapefruit peel waste. The pre-synthesized, nano-crystalline Al_2_O_3_ NPs were characterized by using spectroscopic (UV–vis, FTIR, XRD, and EDX) and microscopic (SEM and TEM) techniques. The formed Al_2_O_3_ NPs exhibited a pronounced absorption peak at 278 nm in the UV–vis spectrum. The average particle size of the as-prepared Al_2_O_3_ NPs was evaluated to be 57.34 nm, and the atomic percentages of O and Al were found to be 54.58 and 45.54, respectively. The fabricated Al_2_O_3_ NPs were evaluated for antioxidant, anti-inflammatory, and immunomodulatory properties. The Al_2_O_3_ NPs showed strong antioxidant potential towards all the four tested assays. The anti-inflammatory and immunomodulatory potential of Al_2_O_3_ NPs was investigated by measuring the production of nitric oxide and superoxide anion (O_2_^•−^), as well as proinflammatory cytokines tumour necrosis factor (TNF-α, IL-6) and inhibition of nuclear factor kappa B (NF- κB). The results revealed that Al_2_O_3_ NPs inhibited the production of O_2_^•−^ (99.4%) at 100 μg mL^−1^ concentrations and intracellular NO^•−^ (55%), proinflammatory cytokines IL-6 (83.3%), and TNF-α (87.9%) at 50 μg mL^−1^ concentrations, respectively. Additionally, the Al_2_O_3_ NPs inhibited 41.8% of nuclear factor kappa B at 20 μg mL^−1^ concentrations. Overall, the outcomes of current research studies indicated that Al_2_O_3_ NPs possess anti-inflammatory and immunomodulatory properties and could be used to treat chronic and acute anti-inflammatory conditions.

## 1. Introduction

Nanotechnology is a multidisciplinary research area that has opened up revolutionary development in the scientific world as scientists can independently control and manipulate atoms as well as molecules [1]. Metal and metal oxide nanoparticles are an emerging group of nanomaterials due to their unique physicochemical characteristics and vast application in various scientific fields, including biomedicine [2], tissue engineering [3], biosensing technology [4], food packaging [5], catalysis [6], nanoelectronics [7], nanorobotics [8], and environmental sciences [9]. Recently, aluminum oxide nanostructures have been a subject of considerable research interest to the scientific community due to their unique optical, electronic, piezoelectric, and biomedical properties [10]. They find potential applications in pharmaceutics, photochemical products, ceramics, paints, additives, and catalysis [11,12]. The newest nanomaterial to grab the attention of scientists is aluminum oxide (Al_2_O_3_) nanoparticles, which have been used for numerous industrial and biological purposes due to their unique features, including large surface area, high mechanical strength, high hardness, good thermal and chemical stability, efficient interaction with metals, and enhanced catalytic activity [13,14,15,16]. Aluminum oxide (Al_2_O_3_) nanoparticles have shown various biomedical applications, including antibacterial [17], antioxidant [18], anticancer, and antipathogenic properties; protein binding [19]; and wound healing [20].

Various strategies for the preparation of Al_2_O_3_ nanomaterials have been described in literature, such as sol-gel [21], sputtering [22], pyrolysis [23], hydrothermal reactions [24], laser ablation [25], and ball milling [26]. However, all these conventional approaches require the use of high energy, pressure, temperature, and hazardous chemicals from various toxic by-products, which are a huge threat to the environment [27]. Consequently, the green synthesis route for the production of Al_2_O_3_ nanoparticles (Al_2_O_3_ NPs) using natural products (plants, sponges, cyanobacteria, and fungus extracts) has captured the enormous interest since natural resources are toxicants free, cost-efficient, convenient, and eco-friendly [28]. Moreover, natural capping and reducing agents are readily provided by these natural sources [29].

Nowadays, the disposal of organic waste control is a rising concern for environmental safety and legislation. Unwanted peels of various fruits such as orange, lemon, grapefruit, pomelo, and pomegranate; vegetable peels; and shells of eggs and shrimp peels, in addition to synthetic organic constituents of solid waste disposal from the municipality, could be utilized in the emerging subject of nanotechnology [30]. For instance, in the process of the extraction of citrus juices (orange, lemon, and grapefruit), around 60–70% of the fruit waste processed is converted to wastes: peels, seeds, and membrane remain [31]. Thus, out of a huge quantity, a lump portion of citrus produced annually is often managed poorly and propounds severe waste consequences on the environment [32]. The recycling of the waste of citrus peels from the industry has the dual intent of waste control and material recycling to produce useful products while keeping the environment free from harmful consequences generated as a result of the accumulation of these wastes [33]. Citrus fruits and peels contain a significant number of bioactive components, including phenol, flavonoids, limonoids, tannins, carotenoids, coumarins, saponins, alkaloid, amino acid, and protein [34,35]. Numerous studies in the literature have addressed the utilization of waste peels of citrus fruits for the preparation of silver [36], titanium oxide [37], iron oxide [38], zinc oxide [39], and zirconium nanoparticles [40]. However, there are only few reports on the synthesis of metallic nanoparticles using peel grapefruit and its biological activities. Faghihi et al. reported the preparation of silver nanoparticles and their effect on damping off caused by *Rhizoctonia solani* in cucumber plants [41]. Another study conducted by Arsene et al. showed the antibacterial potential of silver nanoparticles prepared from grapefruit peels [42]. A recent study reported the synthesis of copper nanoparticles and silver–magnesium oxide bimetallic nanocomposite using the peels of grapefruit [43,44]. To the best of our knowledge, studies related to preparation of Al_2_O_3_ NPs from the waste peels of grapefruit have not yet reported. The peels of grapefruit have been reported to contain terpenes, flavonoids, phenols, tannins, amino acids, and proteins that possess various biological properties [45].

Herein, the current investigation was aimed to prepare Al_2_O_3_ NPs using the waste peels of grapefruit. The formed Al_2_O_3_ NPs were identified and characterized by different spectroscopic and microscopic analyses. The biogenic pre-synthesized nanoparticles were evaluated for antioxidant, anti-inflammatory, and immunomodulatory potential. The Al_2_O_3_ NPs were also evaluated for their photocatalytic potential activity.

## 2. Material and Methods

### 2.1. Cell Lines

The RAW 264.7 mouse macrophages (ATCC^®^TIB-71™, American Type Culture Collection, Manassas, VA, USA) and L929 fibroblast (ATCC^®^ CCL-1™, Thermo Fisher Scientific, Waltham, MA, USA) cell lines used were obtained from subcutaneous, adipose, and areolar tissues of rats. Cells were grown in Dulbecco’s modified Eagle’s medium supplemented with streptomycin (100 μg mL^−1^, Sigma-Aldrich, Hamburg, Germany), 10% of foetal bovine serum (FBS, Sigma-Aldrich, Hamburg, Germany), and penicillin (100 IU mL^−1^, Sigma-Aldrich, Hamburg, Germany) in a humidified atmosphere with a constant supply of 5% CO_2_ at 37 °C.

### 2.2. Botanical Material

Fresh grapefruits were purchased from a local supermarket in Riyadh, Saudi Arabia, in November 2021. The fruits were washed with distilled water and dried by wiping with clean muslin cloth. The peels were separated, dried, and powdered with a domestic blender into fine particles. The powdered peels were directly subjected to the extraction.

### 2.3. Preparation of Grapefruit Peel Extract

The air-dried, powdered grapefruit peels (50 g) were soaked in 250 mL of methanol (98.9%) and subjected to vigorous stirring for 48 h at room temperature. The extract was then filtered by vacuum filtration through Whatman filter paper No. 1 followed by centrifugation at 15,000 rpm for 5 min and finally decanted to obtain clear organic extract. The extraction procedure was repeated three more times under similar conditions. All the filtrates were pooled and freed from organic solvent on a rotary evaporator at 50 ± 5 °C to obtain yellow methanol extract residue (6.89 g). The freshly prepared extract was used for further experiments. 

### 2.4. Preparation of Al_2_O_3_ Nanoparticles Using Grapefruit Extract

The preparation of Al_2_O_3_ NPs was performed using the methanol extract of grapefruit by obeying a previously described method [46]. For the production of Al_2_O_3_ NPs, grapefruit peel extract was utilized as a reducing agent for aluminum nitrate salt. Aluminium nitrate with a molecular weight of 375.13 g/mol served as a precursor for this biosynthesis and was taken in 2 molar quantities and dissolved in distilled water. Briefly, 5 g of grapefruit extract dissolved in 40 mL of DMSO was introduced to 20 mL of an aqueous solution of aluminum nitrate (1.0 × 10^−3^ mol L^−1^) into a 500 mL capacity conical flask under constant magnetic stirring at room temperature for 1 h. The formation of brownish yellow precipitates indicates the formation of Al_2_O_3_ NPs. Afterwards, the reaction mixture was kept undisturbed for 24 h at ambient temperature for the complete settling down of nanoparticles. The reaction mixture was then centrifuged at 10,000 rpm for 10 min, and isolated precipitates were washed with deionized water, followed by thrice with methanol to be free from un-interacted organic impurities. Finally, the purified Al_2_O_3_ NPs were calcinated in a muffle furnace for 1 h at 800 °C.

### 2.5. Photocatalytic Efficiency Measurements

The photocatalytic efficiency of Al_2_O_3_ NPs was tested with methylene blue (MB) and metanil yellow (MY) dye by the photodecomposition method. Briefly, different concentrations (5.0, 10.0, 15.0, and 20.0 ppm) of each dye was mixed with varied concentrations (5, 10, 15, and 20 mg) of as-synthesized Al_2_O_3_ NPs and aerated for 2 h under dark, visible, and UV irradiation conditions. The degradation kinetics were periodically investigated by collecting test samples (3 mL) in 20 min intervals and then centrifuged. The absorption spectra of initial and final dye concentrations were recorded by UV–Vis spectroscopy and the decomposition efficacy were determined by following equation:

$$\mathrm{Degradation}\;\mathrm{efficiency}=\frac{C_{o}-C_{t}}{C_{o}}\;\times \;100$$

where *C_o_* and *C_t_* represent the initial and final concentrations, respectively.

### 2.6. Antioxidant Activity

The antioxidant activity of grapefruit extract and pre-synthesised Al_2_O_3_ NPs was investigated by applying four assays, i.e., a measurement of total antioxidant capacity (TAC), ferric reducing/antioxidant power (FRAP), 1,1-diphenyl2-picrylhydrazyl (DPPH), and 2, 20-azino-bis [3-ethyl benzo thiazoline-6-sulphonic acid] (ABTS) free radical scavenging assays.

#### 2.6.1. Estimation of Total Antioxidant Capacity (TAC)

Total antioxidant capacity of test samples was evaluated by using the previously described Aliyu et al.’s method with slight modifications [47]. Briefly, TAC reagent was prepared by mixing sodium phosphate (28 mm), sulfuric acid (0.6 M), and ammonium molybdate (4 mM) in 50 mL of distilled water. The prepared TAC reagent (900 mL) was then treated with each test sample (100 mL) individually in a conical flask and incubated for 2 h at 90 °C in a water bath. After cooling the reaction mixture, the absorbance was measured at 630 nm by using a micro plate reader. The quantity of gram equivalents of ascorbic acid was applied to measure the total antioxidant activity. Ascorbic acid (1000, 500, 250, 125, 62.5, and 31.25 g/mL) was mixed with methanol to create the calibration curve.

#### 2.6.2. Antioxidant Activity Estimation by Ferric Reducing/Antioxidant Power (FRAP) Assay

The FRAP assay was performed to test the antioxidant effect of Al_2_O_3_ NPs by obeying the modified method of Elya and Noviani (2020) [48]. Briefly, FRAP reagent was prepared by treating 10 parts of sodium acetate buffer (300 mM, pH 3.6), 1 part of FeCl_3_ hexahydrate (20 mM), and 1 part of TPZT (10 mM). Then, 0.2 mL aliquots of three different concentrations (0.5, 1.0, and 2.0 mg mL^−1^) of Al_2_O_3_ NPs were mixed with 3.8 mL of FRAP reagent and incubated at 37 °C for 30 min. The increased absorbance at 593 nm was recorded using a UV-30 spectrophotometer (GIORGIO-BORMAC SRL, Carpi, Italy). The blank used for comparison was prepared by dissolving Al_2_O_3_ in methanol. The results were represented in milligram equivalents of FeSO_4_ per milligram of dry weight. The calibration curve was established using 0.0025, 0.005, 0.01, and 0.02 mg mL^−1^ concentrations of FeSO_4_.

#### 2.6.3. Estimation of Antioxidant Potential by the 2,2-Diphenyl-1-picrylhydrazyl (DPPH) Radical Scavenging Assay

The antioxidant effect of biosynthesized Al_2_O_3_ NPs towards DPPH was estimated by applying the proposed method of Gonzalez-Palma (2016) [49]. In brief, a 1 × 10^−4^ M of methanolic dilution of DPPH was prepared. Then, 1 mL of Al_2_O_3_ NPs at three different concentrations (0.5, 1, and 2 mg mL^−1^) was prepared and treated with 2 mL of DPPH methanolic dilution and placed in the dark for 16 min at ambient temperature. After 16 min dark treatment, the absorbance at 517 nm was noted for the reaction mixture using UV-30 spectrophotometer. The DPPH methanolic dilution was used as the blank and quercetin as the reference standard. The obtained results were represented as milligram equivalents of quercetin per milligram of dry weight. The calibration graph was established by using six different concentrations (0.001, 0.002, 0.005, 0.01, 0.02, and 0.04 mg mL^−1^) of quercetin.

#### 2.6.4. Antioxidant Activity Determination by the ABTS Free Radical Scavenging Assay

The antioxidant potential of Al_2_O_3_ NPs was also evaluated by ABTS assay by following a modified earlier-described method [50]. Briefly, the ABTS^•+^ radical was prepared by the oxidation of ABTS with potassium persulfate. Then, 10 mL of ABTS (7 mM) was treated with 10 mL of potassium persulfate (4.95 mM) and incubated at ambient temperature at 16 h under dark condition. Afterwards, the dilution of the reaction mixture was carried out with methanol until it reached 1–1.5 at 734 nm absorbance values; 0.1 mL aliquots of Al_2_O_3_ NPs at three different concentrations (0.5, 1, and 2 mg mL^−1^) were reacted with 3.9 mL of the ABTS^•+^ solution. The decrease in absorbance at 734 nm was recorded on a spectrophotometer. The ABTS^•+^ solution and quercetin were applied as blank and reference standards. The obtained results were represented in milligram equivalents of quercetin per milligram of dry weight. The calibration curve was plotted by using six (0.00062, 0.00125, 0.0025, 0.005, 0.01, and 0.032 mg mL^−1^) concentrations of quercetin. Two replicates of each experiment were conducted.

### 2.7. Cytotoxicity

The in vitro cytotoxicity of grapefruit extract and pre-synthesised Al_2_O_3_ NPs was measured by the MTT calorimetric method proposed by Hussain et al. (1993) with slight modifications [51]. RAW264.7 macrophages, L929 fibroblast, and MV3 melanoma cell lines were used for the cytotoxicity analysis. Briefly, 96-well plates were individually seeded with 1 × 10^4^ cells/well and incubated overnight at 37 °C with a continuous flow of CO_2_. After incubation, cells were treated with different concentrations (7.5–480 μg mL^−1^) of test sample and positive control doxorubicin (12 μM) and further incubated for one complete day. Afterward, 120 μL of 3-(4,5-dimethylthiazol-2yl)-diphenyl tetrazolium bromide (MTT, 1 mg mL^−1^) reagent was added onto each well plate and incubated at 37 °C for an additional 2 h. DMSO was used to dissolve the formed formazan crystals. The experiments were conducted in triplicates (n = 2), and the results were demonstrated as viability percent.

#### 2.7.1. Quantification of Nitric Oxide (NO)

The in vitro indirect estimation of nitric oxide was conducted by determining the effect of nitric acid production in LPS-activated RAW 264.7 macrophage cells [52]. In brief, 96-well plates were plated with RAW 264.7 macrophage cells at 1.2 × 10^4^ cells/well density and incubated for 24 h at 37 °C with a constant flow of CO_2_. The cells were then reacted with different concentrations (2–120 μg mL^−1^) of test samples (grapefruit extract and Al_2_O_3_ NPs) and incubated for 1 h. Afterward, the treated cells were exposed to 1 μg mL^−1^ of LPS and incubated further for a 24 h. After the 24 h incubation, supernatant of cell was used for the quantification of nitric oxide using Griess reagent [53]. The absorbance was noted at 540 nm on a Multi-mode microplate reader (Max F5 Filter, Molecular Devices Spectra, CA, USA). The positive control applied was L-NIL (50 μg mL^−1^). The experiments were carried out in triplicates, and the results were presented as mean ± SD of concentration (μM) of nitric oxide.

#### 2.7.2. Reduction of Superoxide Anion Production

Superoxide assay was performed to evaluate the inhibitory effects of grapefruit extract and pre-synthesized Al_2_O_3_ NPs on the production superoxide radical (O_2_^•−^) in LPS-activated RAW 267.7 macrophages [54]. Then, 96-well plates were seeded with macrophages at 1.2 × 10^4^ cells/well density and incubated at 37 °C with 5% CO_2_ supply for 24 h. Cells were then exposed to different concentrations (2–120 μg mL^−1^) of samples for 1 h followed by treatment with LPS (1 μg mL^−1^) for 24 h. After 24 h, the supernatant of cell was removed, and cells were treated with NBT (1 mg mL^−1^) and incubated for 2 h. Finally, cells were washed twice with methanol, and formazan crystals formed were solubilized in DMSO and KOH. The absorbance was recorded at 630 nm on a microplate reader. The positive control applied was tempol (12.5 mM). The results were presented as mean ± SD of the production percentage of superoxide anion.

#### 2.7.3. Preventive Effect against Oxidative Damage in RAW 264. 7 Caused by H_2_O_2_

The protective effect of the grapefruit extract and Al_2_O_3_ NPs towards oxidative damage in macrophages caused by hydrogen peroxide (H_2_O_2_) was estimated by modifying the hydrogen peroxide procedure [55]. RAW 264.7 macrophages at a 1.2 × 10^4^ density cells/well were plated in 96-well plates for 24 h. The cells were then treated with different concentrations (1–100 μg mL^−1^) of samples and incubated for 30 min at room temperature. Afterwards, 0.75 mM of H_2_O_2_ was added and incubated for another 2 h. Finally, cell viability was analysed by the MTT colorimetric method. The experiments were conducted in triplicate (n = 2), and the results were represented as mean ± SD of the cellular viability percentage.

#### 2.7.4. Determination of Cytokine In Vitro

The effect of grapefruit extract and Al_2_O_3_ NPs on the levels of cytokine in the macrophage RAW 264.7 were quantified by obeying a modified method of Tian et al. (2021) [56]. Briefly, RAW 264.7 macrophage culture was loaded in 96-microwell plates at a 1.2 × 10^4^ cells/well density and incubated for one complete day (37 °C, 5% CO_2_). After incubation, cells were treated with varied concentrations (1.0–100.0 μg mL^−1^) of test samples (grapefruit extract and Al_2_O_3_ NPs), followed by stimulation with 1 μg mL^−1^ of LPS, and incubated for another 24 h. Afterwards, cell supernatant was freed and applied for the quantification of cytokines (IL-6 and TNF-α) using an enzyme-linked immunosorbent assay (ELISA) kit, with specific standards and antibodies for each tested cytokine as per the manufacturers’ instructions (eBioscience^®^ and Invitrogen^®^, ThermoFisher Scientific, Waltham, MA, USA). The absorbance was noted at 450 and 570 nm in a microplate reader (Filter Max F5, Multi-Mode Microplate Reader, Molecular Devices Spectra, Sunnyval, CA, USA). The reference standard applied was gallic acid (10 μg mL^−1^). Triplicate (n = 2) experiments were conducted, and results were represented in pg/mL.

#### 2.7.5. Determination of Nuclear Factor Kappa B Effect

The ability of grapefruit extract and Al_2_O_3_ NPs to inhibit NF-kB through luciferase expression was evaluated by following Marques et al. (2019) [57]. Embryonic HEK 293 human kidney cells co-cultured with NF-κB luciferase gene was cultured in 96-microwell plates at a 1 × 10^4^ cells/well density and incubated for 48 h. After 48 h incubation, 20 μg mL^−1^ of each test sample (grapefruit extract and Al_2_O_3_ NPs) was added to each well individually followed by the addition of TNF-α (2–0.5 ng mL^−1^ per well) and incubated further for 6 h. Promega Luc assay kit (Madison, WI, USA) was applied to perform the luciferase assay by obeying the instructions of the manufacturer. A microplate luminescence reader (Filter Max F5, Mults-Mode, Molecular Devices Spectra, Sunnyval, CA, USA) was used to monitor the luciferase activity. The results were presented as the percentage inhibition of NF-kB activity. NF-kB inhibition control applied was TPCK (4 μM). Cell viability was investigated in parallel by applying Sulforhodamine B (SRB) assay under similar experimental conditions.

#### 2.7.6. Sulforhodamine B Determination

The Sulforhodamine B (SRB) procedure was applied to estimate the cell viability [58]. Briefly, 96-well plates were loaded with human embryonic kidney cells (HEK 293) and cultured for 48 h at ambient temperature. Afterwards, 20 μg mL^−1^ of each sample (grapefruit extract and Al_2_O_3_ NPs) was poured individually to each well plate and incubated further for 6 h. The cells were then immobilized by adding 20% of trichloroacetic acid (TCA) for 30 min at 4 °C, followed by the addition of SRB solution (0.4% SRB in 18% acetic acid), and incubated for another 30 min. The plate was then washed with 1% acetic acid and dried. Finally, 10 mM of Tris base solution (pH, 10) was used to dissolve protein-bound dye, and absorbance was recorded at 515 nm on a spectrophotometer. The experiments were repeated thrice (n = 3), and the results were presented as survival percent.

### 2.8. Statistical Analysis

All the experiments were performed in triplicate. GraphPad Prism 5 was used to conduct statistical analysis (San Diego, CA, USA). The information was reported as a mean standard deviation (SD). Univariate analysis of variance (ANOVA) and Tukey’s post hoc tests were used to make statistical comparisons. A *p* < 0.05 was considered statistically significant by using 18.0. Software, IBM SPSS Modeler (Agilent, Santa Clara, CA, USA). The SD error for each test sample was presented by error bars on monographs.

## 3. Results and Discussion

An eco-friendly biosynthesis of Al_2_O_3_ nanoparticles using grapefruit extract as the reducing agent at an optimized pH (7.4) was envisaged by the colour change of the reaction mixture from yellow to dark brown. The confirmation of Al_2_O_3_ NPs reduction was designated by the conversion of yellow coloured reaction solution to dark brown. The obtained Al_2_O_3_ NPs were free from nitrate ions in deionized water and dried by calcination treatment. The resulted product Al_2_O_3_ NPs were verified by spectroscopic (UV–vis, FTIR, XRD) and microscopic (SEM, EDX, TEM) techniques.

### 3.1. Characterization of Al_2_O_3_ Nanoparticles

**Figure 1:**
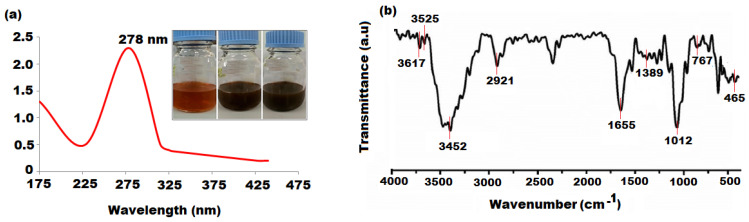
(**a**) UV–vis and (**b**) FTIR spectra of biosynthesized Al_2_O_3_ nanoparticles at 278 nm and 4000–500 cm^−1^.

The capability of the UV absorption of Al_2_O_3_ NPs is associated with bandgap energy and was determined by UV–vis spectrum. The spectrum of as-synthesized Al_2_O_3_ NPs exhibited an absorption peak at 278 nm in the UV region, as depicted in Figure 1a. The Wood and Tauc procedure was applied to estimate the bandgap energy (Eg) by obeying the (hνα) = (hν − Eg)n equation, where α, h, ν, Eg, and n represent the absorption coefficient, Planck’s constant, frequency, absorption bandgap energy, and constant electronic transitions. The bandgap absorption energy value obtained from grapefruit peel mediated-Al_2_O_3_ NPs was 3.31 eV and is inconsistent with the reported literature [59]. The FTIR analysis of pre-synthesized Al_2_O_3_ NPs before annealing was conducted to identify different functional groups’ moieties participating in the reduction, stabilization, and capping of Al_2_O_3_ NPs. Figure 1b displays the FTIR spectrum of biosynthesized Al_2_O_3_ NPs showing strong peaks at 3617 cm^−1^, 3525 cm^−1^, and 3452 cm^−1^ due to the symmetrical stretching vibration of hydroxyl (OH), methyl (-CH_3_), and methoxy (-OCH_3_) groups, respectively. The bending vibration peaks, appearing at 1389 cm^−1^ and 1012 cm^−1^, correspond to hydroxyl (OH) and carboxylate (RCOO^−^) groups. Two small peaks originating at 767 cm^−1^ and 465 cm^−1^ indicate the presence of alkyl and amide groups in the as-synthesized Al_2_O_3_ NPs. The FTIR results of Al_2_O_3_ NPs confirm the existence of polyhydroxyl groups from the grapefruit peel bound to Al_2_O_3_ NPs [60]. These results were in good agreement with available studies on the preparation of silver, magnesium, copper, and gold nanoparticles using grapefruit peel extract [61,62,63]. The FTIR analysis of pre-synthesized Al_2_O_3_ NPs after annealing has been performed and included as Supplementary Information (Figure S1).

**Figure 2:**
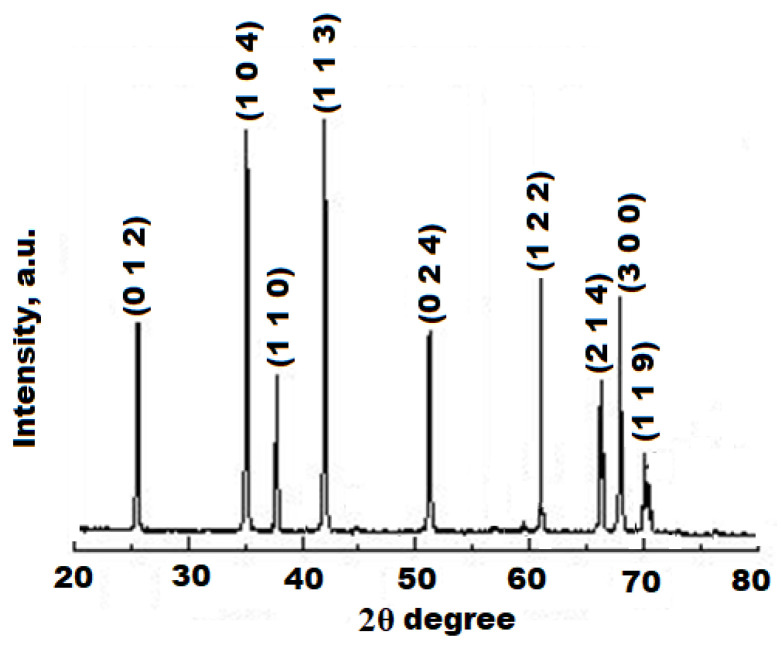
XRD spectrum of biosynthesized Al_2_O_3_ nanoparticles.

The XRD pattern of as-synthesized, nano-sized Al_2_O_3_ NPs showed a rhombohedral structure, which correspond and coordinate the catalogue of the JCPDS standard file no: 71-1683. The 2θ values were obtained: 25.1°, 34.3°, 37.8°, 42.8°, 51.9° 61.2°, 65.9°, 68.1°, and 70.3° and correspond to (0 1 2), (1 0 4), (1 1 0), (1 1 3), (0 2 4), (1 2 2), (2 1 4), (3 0 0), and (1 1 9). The average size of Al_2_O_3_ NPs was determined to be 57.34 nm across all peaks. The Debye Scherrer equation, D = 0.9λ/βcosθ, yielded a lattice constant of 0.625, where D, λ, β, and θ signify crystalline size, wavelength of CuKα radiation, full width half maximum, and Bragg’s angle of X ray diffraction peak, respectively (Figure 2). The most stable phase of Al_2_O_3_ was obtained at 800 °C in the XRD analysis.

**Figure 3:**
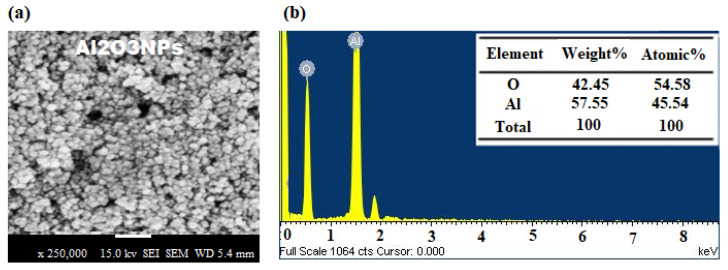
(**a**) SEM and (**b**) EDX images of biosynthesized Al_2_O_3_ nanoparticles.

**Figure 4:**
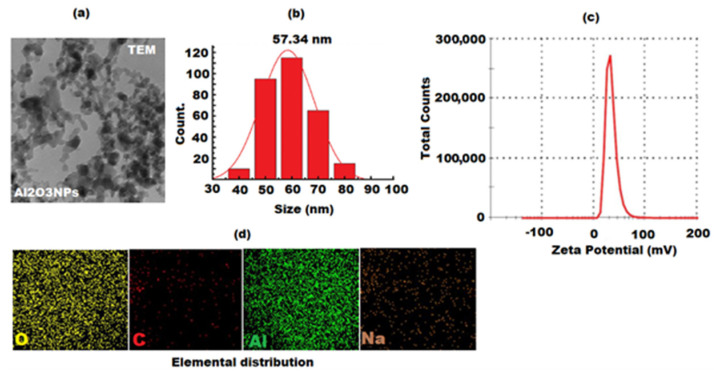
(**a**) TEM, (**b**) particle size distribution, (**c**) DSL analysis, and (**d**) Elemental distribution of oxygen (O), aluminum (Al), carbon (C), and sodium in the EDX mapping of Al_2_O_3_ nanoparticles.

The morphological surface of formed Al_2_O_3_ NPs was visualized by a scanning electron microscope (SEM) and transmission electron microscope (TEM) under different magnifications. The SEM images of Al_2_O_3_ NPs display hexagonal structures under ×250,000 and ×150,000 magnifications (Figure 3a). The variation in surface morphology was attributed to intermolecular interaction, lattice mismatch, and the existence of residual oxides [64]. The purity of Al_2_O_3_ NPs was demonstrated by EDX analysis, which only detects aluminum and oxygen. The average atomic% of O and Al was found to be 54.58 and 45.54, respectively (Figure 3b). However, the TEM was applied to confirm the size and morphological shape of the as-synthesized Al_2_O_3_ NPs as well as the surface attachment of grapefruit peel extract (Figure 4a,b). The results of TEM images showed that most of the formed Al_2_O_3_ NPs were hexagonal and spherical in shape with 50–100 nm average size range (Figure S2). However, some nanoparticles were agglomerated to form non-spherical structures that have resulted in bigger particle sizes [65]. It was also noticed that the characteristics of biosynthesized Al_2_O_3_ NPs might be influenced by the phytoconstituents present in the grapefruit peel extract.

The particle size distribution and zeta potential measurements are used to characterize nanoparticles and disclose information about their size distribution, stability, surface charge, and colloidal behaviour [66]. The zetasizer determination of biosynthesized Al_2_O_3_ NPs from grapefruit extract showed that the average value of nanoparticles size distribution was 57.34 nm (Figure 4c). However, the average particle size distribution of nanoparticles supports the TEM analysis results by bringing the average size values closer to the TEM profile size distribution ranges. Furthermore, the negative zeta potential values indicated the presence of possible capping and stabilization of nanoparticles by biomolecules present in grapefruit peel extract, as well as the presence of strong agglomeration, by keeping the particles apart, which increased the negative repulsion among the particles and thus confirmed a higher stability. Furthermore, elemental mapping measurements and elemental profiles of Al_2_O_3_ NPs revealed the occurrence of four dominant elements (Al, C, O, and Na) (Figure 4d), indicating the Al_2_O_3_ NPs presence in the sample. The peaks appeared in C and O were attributed to the secondary metabolites in the grapefruit peel extract [67].

### 3.2. Photocatalytic Efficiency of Al_2_O_3_ NPs

The photocatalytic degradation performance of biosynthesized Al_2_O_3_ NPs was examined under different reaction conditions. The photocatalytic efficacy of nanoparticles depends on surface area, morphology, particle size, crystallinity, bandgap, and OH^•^ free radical content on the photocatalyst surface [68]. The absorption of light causes the release of electron and holes on the photocatalyst surface, and released electrons and holes will participate in the reaction or reunite. If an extra surface is available for the electrons and holes prior to the reunion, they will relocate, and electrons are trapped by the photocatalyst while the holes are activated to generate OH^•^ and HO^2•^. The ternary structure has more surfaces available for the relocation of photogenerated charge carriers, and the produced hydroxyl (OH^•^ and HO^2•^) free radicals were utilized efficiently to decompose MB/MY dye. The results of UV–vis spectra obtained in this study revealed that pre-synthesized Al_2_O_3_ NPs was active in the UV domain. Different parameters including photocatalyst dose, light source, dye concentration, pH, and irradiation time were systematically explored, and MB was applied as a reference pollutant photocalatytic degradation in this investigation. The variation in intensity of the absorption peak of MB dye noted at 663 nm was monitored to conclude the obtained results.

**Figure 5:**
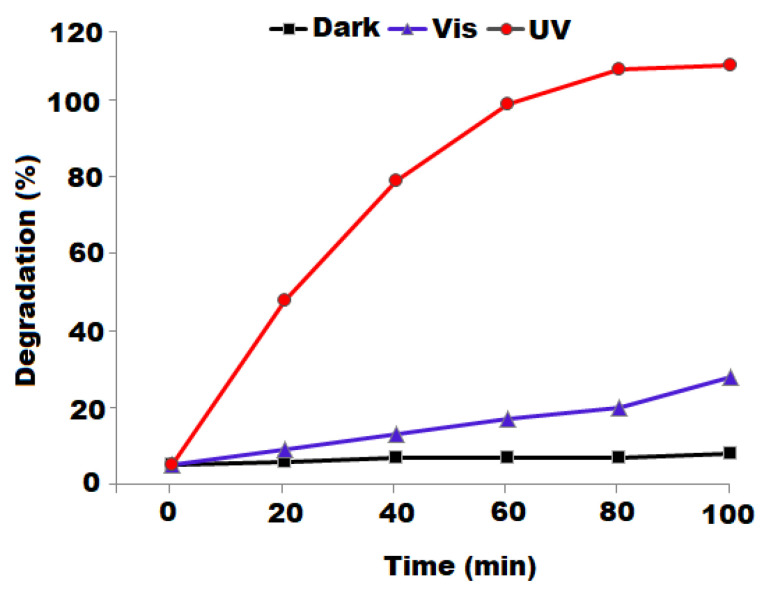
Influence of light source on MB photodegradation employing the biosynthesized Al_2_O_3_ nanoparticles.

The photocatalytic degradation of MB dye in the presence of pre-synthesized Al_2_O_3_ NPs under the influence of light was studied. Three different environments (dark, UV light, and natural solar irradiation) were applied to the reaction mixture containing 5 ppm of MB and 15 mg of Al_2_O_3_ NPs. Under the dark condition, the insignificant degradation of MB dye was observed, while the photodegradation of MB was found to be much higher in the visible light irradiation and UV irradiation (Figure 5). The UV–vis spectra of the as-synthesized Al_2_O_3_ NPs supported the obtained results for the decomposition of MB under UV irradiation. The MB dye showed ~98% of degradation in 100 min under UV irradiation, whereas MY dye exhibited 96% and was noticed under similar conditions. However, around ~22% of degradation was observed in MB/MY dye under visible light in a 100 min time span.

The effect of the dose of photocatalyst (Al_2_O_3_ NPs) on the photodecompostion of MB/MY dye was measured at various concentrations (5–20 mg) in the presence of UV radiation. The results revealed that the dose of photocatalyst considerably influenced the MB/MY photodegradation. It was observed that with the increase in concentration of Al_2_O_3_ NPs (5–15 mg), the rate of decomposition was increased by ~64% to ~98% and ~61% to ~96% for MB and MY dye, respectively (Figure S3). The alleviation in the degradation can be attributed to the available active sites in Al_2_O_3_ NPs that produce more radical ions. However, the further increase in the concentration (20 mg) of photocatalyst has led to a decrease in decomposition efficacy to ~88% of MB dye under similar photocatalytic conditions, while the decomposition efficacy of MY dye was enhanced to 99% with the increase of photocatalyst concentration (20 mg). If the amount of photocatalyst is above the critical boundary, the dispersion of nanoparticles in the solutions gets restricted due to the limited available space, and the particles stick to each other and get aggregated. Thus, most of the active sites of photocatalysis were blocked, and the decomposition efficacy of the system was decreased [69]. Moreover, it was noticed that the degradation of MB and MY was 9% and 8%, respectively, in the absence of photocatalysts under the above applied conditions. Hence, the optimal photocatalyst concentration selected for this experiment was 15 mg and was used in the rest of experiments for the optimization of parameters. The effect of dye concentration and pH has been discussed with supporting information.

### 3.3. Antioxidant Activity

The antioxidant potential of grapefruit extract and Al_2_O_3_ nanoparticles were quantified by the TAC, FRAP, DPPH, and ABTS methods. The antioxidant potential values estimated for each sample correspond to 0.1 mg mL^−1^ concentrations of the investigating samples. Among all the analysed concentrations, this was the selected concentration that persisted in the absorbance values of the pattern for all the applied methods. The data of antioxidant activity of test samples at three different concentrations (0.5, 1, and 2 mg mL^−1^) quantified by four methods were presented in Table 1. The comparison between the assays was facilitated by applying quercetin as a pattern, except for TAC and FRAP assay, which were demonstrated in ascorbic acid and FeSO_4_ equivalents. The results of the total antioxidant activity of grapefruit peel extract and Al_2_O_3_ NPs revealed that Al_2_O_3_ NPs (0.010 AA mg/mg dw) expressed a stronger antioxidant effect in contrast to grapefruit peel extract (0.007 AA mg/mg dw). The antioxidant activity estimated by DPPH in each investigated sample, biosynthesized Al_2_O_3_ NPs, showed the highest value (0.036 QE mg/mg dw) in contrast to grapefruit peel extract (0.019 QE mg/mg dw). This method illustrated a difference of around two times more antioxidant potential for pre-synthesized Al_2_O_3_ NPs with respect to grapefruit peel extract, whereas the quantification of antioxidant effect by ABTS exhibited the highest value for as-synthesized Al_2_O_3_ nanoparticles (0.015 QE mg/mg dw), followed by grapefruit peel extract (0.012 QE mg/mg dw), displaying two times more the effect of Al_2_O_3_ NPs in comparison to grapefruit peel extract. Lastly, the values acquired by FRAP revealed that the moderate effect was that of the grapefruit peel extract (0.041 FeSO_4_ E mg/mg dw), and the highest antioxidant effect corresponded to Al_2_O_3_ NPs (0.091 FeSO_4_ E mg/mg dw), indicating that a 2-flod difference was established between Al_2_O_3_ NPs and grapefruit peel extract. The correlation between the assays used to estimate the antioxidant potential of the Al_2_O_3_ NPs and grapefruit peel extract were analysed (Table 2). A potential correlation was established between the different assays. It is noteworthy that FRAP was the procedure with the least significance levels when compared with the other assays. The TAC, FRAP, DPPH, and ABTS scavenging methods confirmed that the aluminum oxide nanoparticles possess strong antioxidant potential. The presence of functional groups on the surface of aluminum oxide nanoparticles is responsible for these features. The presence of phytochemicals including flavonoids and phenolics with hydroxyl (OH) and phenolic groups on the surface serves as capping agents on these nanoparticles and may account for the observed antioxidant ability.

### 3.4. In Vitro Cytotoxicity

**Figure 6:**
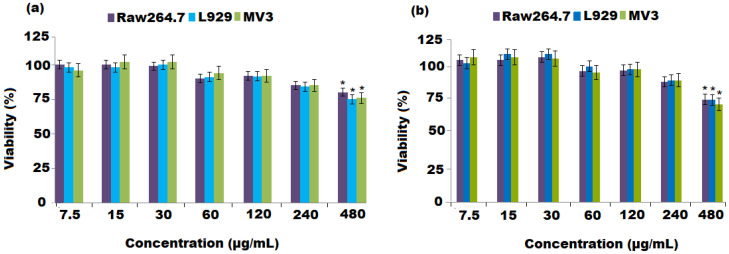
Cellular viability measurements of RAW 264.7, L929, and MV3 cancer cells after 24 h treatment with (**a**) grapefruit peel extract and (**b**) Al_2_O_3_ nanoparticles. Values are represented as cellular viability percentage and expressed as the means ± SD of three independent experiments. * Statistically significant (*p* < 0.05) as compared to the control cells.

The MTT colorimetric assay was applied to measure the cytotoxicities of the grapefruit extract and Al_2_O_3_ NPs in the macrophage (RAW 264.7), fibroblast (L929), and melanoma (MV3) cancer cell lines. The grapefruit extract and Al_2_O_3_ NPs showed no effect on cell viability at concentrations below 480 and 120 μg mL^−1^, respectively (Figure 6a,b). Following the determination of the samples’ non-cytotoxic values, a maximum concentration of 100 μg mL^−1^ was established for use in following cell culture tests. The results showed that Al_2_O_3_ NPs induced a concentration-dependent decrease of cell viability at much lower concentrations was observed. This could be attributed to the positive charge of alminum oxide ions, long alky side chain, and nature of chemical constituents present in grapefruit peel extract have some influence on this toxicity.

#### 3.4.1. Protective Impact towards the Oxidative Damage Caused by H_2_O_2_ in RAW 264.7 Macrophages

**Figure 7:**
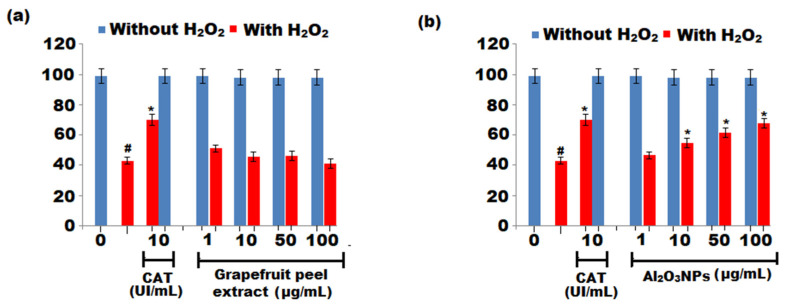
Protective effect of (**a**) grapefruit peels extract and (**b**) Al_2_O_3_ NPs in RAW 264.7 macrophages towards damage caused by hydrogen peroxide (H_2_O_2_). The results were represented as ± SD (n = 2). ^#^ Significant (*p* < 0.05) and * Significant (*p* < 0.05) compared to negative control without H_2_O_2_ and H_2_O_2_ control by one-way ANOVA.

The protective effects of the grapefruit extract and Al_2_O_3_ NPs towards hydrogen peroxide (H_2_O_2_)-induced cell damage were determined in RAW 264.7 macrophages. The Al_2_O_3_ NPs exhibited a protective effect of 34.5, 45.5, and 71.5% at 10, 50, and 100 μg mL^−1^ concentrations, respectively (Figure 7a,b). The current study found that Al_2_O_3_ NPs, besides lowering the production of NO and O_2_, have the ability to suppress the production of interleukin-6 (IL-6) and tumour necrosis factor-α (TNF-α) pro-inflammatory cytokines, as well as the signalling pathway of the transcription factor NF-κB.

#### 3.4.2. In Vitro Indirect Estimation of Nitric Oxide (NO)

**Figure 8:**
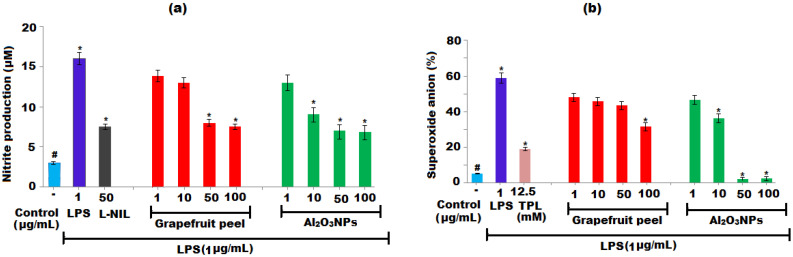
Effect of grapefruit peels extract and Al_2_O_3_ NPs on the production (**a**) nitric oxide (NO) and (**b**) superoxide radical (O_2_^•−^). (**a**) RAW 264.7 macrophages were treated to varied sample concentrations and stimulated with LPS (1 μg mL^−1^) and (**b**) RAW 264.7 macrophages were treated to varied sample concentrations and stimulated with LPS after 30 min. L-NIL and tempol were used as positive control for NO and superoxide radical (O_2_^•−^), respectively. The results were expressed as mean ± SD (n = 2). ^#^ Significant (*p* < 0.05) and * Significant (*p* < 0.05) compared to the negative control without LPS and LPS-induced cells by one-way ANOVA followed by Tukey’s post hoc test.

Reactive oxygen species (ROS) are produced by neutrophils and macrophages during inflammation and other normal cellular metabolic processes. An imbalance favouring free radical creation occurs when the rate of generation of these free radicals is increased or the protective antioxidant system is lowered, resulting in increased oxidative stress and eventual tissue damage. Increased ROS production has been observed in a variety of pathophysiologies as well as other systemic problems. Several investigations have shown that both NO and O_2_^•−^ are involved in a variety of pathophysiological processes and are thus regarded important therapeutic targets. The indirect estimation of nitric oxide (NO) was conducted by determining the effect on production of nitrite in LPS-activated RAW 264.7 macrophage culture. The grapefruit extract and Al_2_O_3_ NPs inhibited the NO production in a dose-dependent manner. Inhibitions of 52% and 55% in the production of NO were noticed after exposure to grapefruit extract and Al_2_O_3_ NPs at 50 μg mL^−1^, respectively (Figure 8a). The specific inhibitions I-NOS and L-NIL were applied as the positive control at 50 μg mL^−1^, leading to a 49.5% inhibition of NO production.

#### 3.4.3. In Vitro Superoxide Estimation

The results of in vitro superoxide production revealed that the Al_2_O_3_ NPs inhibited 31.5, 98.7, and 99.4% of superoxide radical (O_2_^•−^) generation in the LPS-activated RAW 264.7 macrophage culture at concentrations of 10, 50, and 100 μg mL^−1^, respectively, in a dose-dependent manner, while the grapefruit extract showed a 52.9% inhibition of superoxide radical (O_2_^•−^) production at 100 μg mL^−1^. However, positive control tempol (12.5) showed a 67.3% inhibition of O_2_^•−^ production, as displayed in Figure 8b. Thus, the findings of the study demonstrated that the Al_2_O_3_ NPs have a detrimental influence on the formation of nitric oxide and superoxide anions in the LPS-stimulated macrophage culture. These findings point to significant biological activity that contributes to the regulation of oxidative stress and, as a result, the inhibition of the inflammatory response. Several investigations have shown that both NO and O_2_^•−^ are involved in numerous pathophysiological processes and are thus the important therapeutic targets [70,71].

#### 3.4.4. Estimation of Cytokine Production

**Figure 9:**
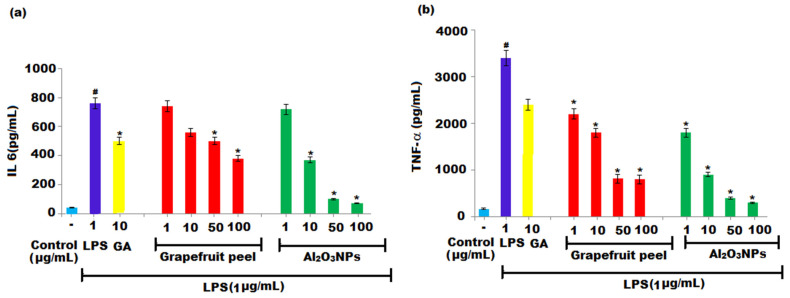
Effect of grapefruit peels extract and Al_2_O_3_ NPs on the levels of (**a**) IL-6 and (**b**) TNF-α proinflammatory cytokines. RAW 264.7 macrophages were treated with varied concentrations of samples in the presence or absence of LPS. The ^#^ Significant (*p* < 0.05) and * Significant (*p* < 0.05) compared to the negative control without LPS and LPS-induced cells, respectively, by one-way ANOVA followed by Tukey’s post hoc test.

Two key pro-inflammatory cytokines, IL-6 and TNF-α, play an important role in inflammatory diseases. The supernatant of cells was exposed to varying concentrations of grapefruit extract, and Al_2_O_3_ NPs was applied for the estimation of cytokines (IL-6 and TNF-α). The results revealed that the grapefruit extract exhibited an inhibition of IL-6 production by 41% and 46% at 50 and 100 μg mL^−1^ concentrations, respectively (Figure 9a), whereas the production of TNF-α was inhibited by 32.3, 49.5, 83.5, and 72.4% at concentrations of 1, 10, 50, and 100 μg mL^−1^, respectively, after grapefruit extract treatment (Figure 9b). However, the production of IL-6 was inhibited by 83.3% and 86.7%, and TNF-α production was inhibited by 87.9 and 91.6%, at 50 and 100 μg mL^−1^ concentrations. In carrageenan-injected rat paws, Al_2_O_3_ NPs potentially reduced TNF-α production without influencing IL-1 production. The ability to decrease endotoxin induced in mice, levels of TNF-α, interleukin (IL)-1β, IL-18, interferon (IFN)-γ, and peripheral nitrate/nitrite was attributed to Al_2_O_3_ and the chemical components (flavonoids and phenolics) of grapefruit extract. This study showed for the first time that the Al_2_O_3_ NPs could decrease the production of pro-inflammatory cytokines interleukin-6 (IL-6) and tumour necrosis factor-α (TNF-α). The anti-inflammatory effects of Al_2_O_3_ NPs can be attributed to the inhibition of prostaglandin production or release by aluminum and oxygen ions as well as chemical components present in grapefruit extract, subsequently lowering pro-inflammatory TNF-α, IL-1, and IL-6 production.

#### 3.4.5. Determination of Nuclear Factor Kappa B Activity

The NF-κB transcription factor is regarded as a critical mediator in the human immune system. The NF-κB signalling system controls the expression of several genes implicated in inflammatory responses, including proinflammatory cytokines, adhesion molecules, chemokines, and inducible enzymes such as ions [72]. Thus, the inhibition of the NF-κB signalling pathway has been explored widely as an effective important therapeutic approach for the cure of various malignant inflammatory disorders [73]. The anti-inflammatory potential of the grapefruit extract and Al_2_O_3_ NPs was investigated by luciferase reporter assay by quantifying the NF-κB inhibition. The human embryonic kidney (HEK 293) cells were used and treated with test 20 μg mL^−1^ of samples (grapefruit extract and Al_2_O_3_ NPs). The grapefruit extract and Al_2_O_3_ NPs showed no cytotoxic effect on the renal cells, exerting survival rates of 92.7 ± 2.1% and 96.5 ± 3.0%, respectively, as measured by SRB assay. Additionally, they exhibited potent inhibitions of 32.9 ± 1.6% and 41.8 ± 10.5% of the NF-κB effect at the same concentration. The results of this study suggested that the signalling pathway of NF-κB is partially involved in the possible molecular mechanism in which Al_2_O_3_ NPs inhibit the oxidative stress and pro-inflammatory mediator’s expression.

## 4. Conclusions

Eco-friendly, biogenic aluminum oxide nanoparticles (Al_2_O_3_ NPs) were prepared using grapefruit peel waste extract by using a simple approach. The formed nanoparticles were identified and confirmed by different analytical techniques such as UV–vis, FTIR, SEM, EDX, and TEM. The average particle size of crystalline Al_2_O_3_ NPs was around 10–60 nm. The SEM, TEM and FTIR images of Al_2_O_3_ nanoparticles confirmed the spherical shape with homogenous agglomeration and the existence of functional groups in the Al_2_O_3_ NPs. The pre-synthesized biogenic nanoparticles expressed were excellent for antioxidant, anti-inflammatory, and immunomodulatory potentials compared to grapefruit peel extract. The outcome of the study favours the utilization of Al_2_O_3_ NPs as an antioxidant and for the cure of inflammation. The formed Al_2_O_3_ nanoparticles have shown an incredible ability to decrease the production of pro-inflammatory cytokines interleukin-6 (IL-6) and tumour necrosis factor-α (TNF-α), as well as the signalling pathway of the transcription factor NF-B, in addition to lowering NO and O_2_ generation. In conclusion, this investigation showed that Al_2_O_3_ NPs could be investigated as a valuable source of new and effective anti-inflammatory agents. The pre-synthesized Al_2_O_3_ NPs showed remarkable therapeutic potential for modulating and regulating macrophage activation and could be used to treat a number of inflammatory disorders.

## Figures and Tables

**Table 1:** The antioxidant activity of grapefruit peel extract and Al_2_O_3_ NPs by TAC, DPPH, ABTS, and FRAPS assays.

Sample	TAC	DPPH	ABTS	FRAP
	(AA mg/mg dw)	(QE mg/mg dw)	(QE mg/mg dw)	(FeSO_4_E mg/mg dw)
Grapefruit peel extract	0.007 ± 0.0001	0.019 ± 0.0004	0.012 ± 0.0002	0.041 ± 0.003
Al_2_O_3_ NPs	0.010 ± 0.0005	0.036 ± 0.0006	0.015 ± 0.0005	0.091 ± 0.002

Ascorbic acid (AA), Quercetin (QE), Iron sulphate (FeSO_4_).

**Table 2:** Correlation matrix (Pearson’s correlation coefficients) for the study of grapefruit extract and Al_2_O_3_ NPs.

Method	DPPH	FRAP	ABTS	TAC
TAC	0.932 ***	0.763 **	0.918 ***	
ABTS	0.915 ***	0.726 **		
FRAP	0.768 **			

TAC: Total antioxidant capacity; *** Significant at *p* < 0.001, ** Significant at *p* < 0.01.

## Data Availability

The data supporting the outcome of this study have been incorporated within the manuscript.

## Supplementary Materials

The following supporting information can be downloaded at: https://www.mdpi.com/article/10.3390/nano12111885/s1, Figure S1: FTIR spectra of biosynthesized Al_2_O_3_ nanoparticles at 4000–500 cm^−1^ after annealing; Figure S2: TEM image of Al_2_O_3_ NPs at ×250,000 magnifications; Figure S3: MY photodegradation employing the biosynthesized Al_2_O_3_ nanoparticles.

## Abbreviations

**Name**	**Abbreviation**	**Name**	**Abbreviation**	**Name**	**Abbreviation**
Methanol	MeOH	Dimethyl sulfoxide	DMSO	Aluminum oxide nanoparticles	Al_2_O_3_ NPs
Ascorbic acid	AA	2, 20—azino-bis [3-ethyl benzo thiazoline-6-sulphonic acid]	ABTS	Dimethylformamide	DMF
2,2—Diphenyl-1-picrylhydrazyl	DPPH	Ferric reducing/antioxidant power	FRAP	Dulbecco’s modified Eagle medium	DMEM
Enzyme-linked immunosorbent assay	ELISA	Iron sulfate	FeSO_4_	Foetal bovine serum	FBS
Iron(III) chloride hexahydrate	FeCl_3_.6H_2_O	Hydrogen peroxide	H_2_O_2_	Human embryonic kidney	HEK
Interleukin-6	IL-6	Interferon	IFN	Fibroblast	L929
Lipopolysaccharide	LPS	Methylene blue	MB	Metanil yellow	MY
3-(4,5-dimethylthiazol-2-yl)-2,5-diphenyl-2H-tetrazolium bromide	MTT	Melanoma	MV3	Nuclear factor kappa B	NF-κB
Nitric oxide	NO	Hydroxyl	OH	Quercetin	QE
Macrophage	RAW 264.7	carboxylate	RCOO	Superoxide radical	O_2_^•−^
Standard deviation	SD	Sulforhodamine B	SRB	Tumour necrosis factor	TNF
Tetrahydrofuran	THF	2,4,6-tri(2-pyridyl)-s-triazine	TPZT	Total antioxidant capacity	TAC
Trichloroacetic acid	TCA	Catahein	CAT	Inducible nitric oxide synthase	I-NOS
L-N(6)-(1-iminoethyl)lysine hydrochloride	L-NIL	Dynamic light scattering	DLS	Energy dispersive x-ray	EDX
Scanning electron microscope	SEM	Transmission electron microscopy	TEM	Bandgap energy	Eg
Fourier-transform infrared	FTIR	Ultraviolet-visible	UV-vis	x-ray diffraction	XRD
Zeta potential	ZP	Interleukin-1	IL-1	Methoxy	-OCH_3_
