# Supplementary material for: Grapefruit Extract-Mediated Fabrication of Photosensitive Aluminum Oxide Nanoparticle and Their Antioxidant and Anti-Inflammatory Potential
Source: Nanomaterials (Basel). 2022 May 31;12(11):1885. doi: 10.3390/nano12111885 (PMC9182307; doi:10.3390/nano12111885)
Supplement: Supplementary file 1 [file nanomaterials-12-01885-s001.zip › nanomaterials-1708856-SI.pdf]

Supplementary Materials

Grapefruit Extract-Mediated Fabrication of Photosensitive Aluminum Oxide Nanoparticle and Their Antioxidant and Anti-Inflammatory Potential

Kholoud A. Bokhary [1], Farah Maqsood [1], Musarat Amina [2,*], Amal Aldarwesh [1], Hanan K.Mofty [1] and Hanan M. Al-yousef [2]

[1] Department of Optometry and Vision Science, College of Applied Medical Science, King Saud University, Riyadh 11451, Saudi Arabia; kbokhary@ksu.edu.sa (K.A.B.); fmaqsood@ksu.edu.sa (F.M.); aaldarweesh@ksu.edu.sa (A.A.); hmofty@ksu.edu.sa (H.M.)

[2] Department of Pharmacognosy, College of Pharmacy, King Saud University, Riyadh 11451, Saudi Arabia; halyousef@ksu.edu.sa

* Correspondence: mamina@ksu.edu.sa

Chemicals and reagents

All the chemicals and reagent used in this study, including; methanol (MeOH, 98.9%), dimethyl sulfoxide (DMSO, 99.9%), sodium hydroxide (99.8%) aluminium nitrate nanohydrate ($Al(NO_3)_3.9H_2O$, 99.9%), methylene blue (MB) solution, metanil yellow (MY, 70%), sodium phosphate (≥ 98%), sulphuric acid (93-98%), ammonium molybdate (99.8%) tetrahydrofuran (THF), Iron(III) chloride hexahydrate ($FeCl_3.6H_2O$, ≥ 98%), 2,4,6-tri(2-pyridyl)-s-triazine (TPZT, ≥98%), N, N-dimethylformamide (DMF, 99.0%) 2,2-diphenyl-1-picrylhydrazyn (DPPH, 95%) 2,2'-Azino-bis(3-Ethylbenzthiazoline-6-Sulfonic Acid) (ABTS, > 98%), potassium persulfate (≥ 99.0%), ascorbic acid (≥ 99%), quercetin, doxorubicin, 3-(4,5-dimethylthiazol-2yl)-diphenyl tetrazolium bromide (MTT), modified Eagle's medium (DMEM), fetal bovine serum (FBS), 3-(4,5-dimethylthiazol-2yl)-diphenyl tetrazolium bromide (MTT), streptomycin, penicillin, gallic acid, lipopolysaccharide (LPS), trichloroacetic acid, acetic acid, and were purchased from Sigma Aldrich (Hamburg, Germany).

Characterization of Al_2O_3 NPs

The synthesis of Al_2O_3 NPs was verified by various spectroscopic techniques: UV-Vis spectrum was performed in the 200 to 800 nm absorption wavelength range using spectrophotometer (UV 2450, Shimadzu, Kanagawa, Japan). FTIR spectroscopy analysis was performed to detect and measure the functional group biosynthesized grapefruit- Al_2O_3 NPs in a range of 4000–400 cm^{-1} using KBr pellets (PerkinElmer, Waltham, MA, USA). X-ray diffraction scanning was conducted to determine the crystalline nature of biosynthesized Al_2O_3 NPs within the range of 20-80 θ using diffractometer (D8 Advance, Bruker, Erfurt, Germany). Scanning Electron Microscopy (SEM) equipped with Energy Dispersive X-ray (EDX) spectroscopy (model JSM-7610F, JEOL, USA) was applied to visualize the shape of biosynthesized Al_2O_3 NPs. Transmission electron microscopy (JEM-1230, Japan) analysis was performed for particle size determination Zeta potential (ZP) and dynamic light scattering (DLS) analyzer was used to determine the stability and size of Al_2O_3 NPs (dynapro1Plate Reader III, Waytt, Japan).

Impact of MB/MY concentration

By altering the MB/MY concentration from 5 to 20 ppm under UV irradiation, while keeping the photocatalyst dose at 15 mg, the effect of starting dye concentrations on the degradation performance of the MB/MY was investigated. The results obtained revealed that the photocatalytic performance of Al_2O_3 NPs is inversely related to the concentration of

dye (MB/MY) under identical conditions, i.e., at lowest concentration (5 ppm) of MB/MY, highest degradation efficacy was noticed. The decomposition of MB and MY were declined from ~98% to ~56% and ~95% to ~54% respectively, by increasing MB/MY concentration from 5 to 20 ppm. This reduction is due to a decrease in light absorption on the photocatalyst surface caused by increasing dye concentration, which limits the formation of $OH^{\bullet}$ radical ions, which are important in the photodegradation process.

Impact of pH value

The pH of the solution is well-known to be one of the most important parameters in the photocatalytic decomposition of organic dyes. This effect is related to a shift in surface charge of photocatalyst, which had a significant impact on photocatalytic efficacy. The availability of $OH^{\bullet}$ radicals in the reaction media is usually closely proportional to the photocatalytic performance of the photocatalyst, which enhances the photocatalytic degradation of MB dye in alkaline aqueous solution by many folds. The effect of pH on the photodegradation of MB dye in the presence of Al_2O_3 photocatalyst produced. At three distinct pH values of 4, 7, and 10, the effect of pH on the elimination of MB/MY was investigated. The results demonstrated that increasing the pH value to 10 resulted in increased photodegradation. The lowest degradation performance was found at an acidic pH value (i.e., pH 4), with ~38% and ~33% degradation of MB and MY, respectively. The Al_2O_3 photocatalyst showed displayed maximum degradartion effect as the pH of the solution was increased and nearly 99% degradation of MB was attained at pH 10. This could be due to the production of negative charges on the surface due to the higher pH value. Since MB is a cationic dye with a positive charge, and dyes are adsorbed on surfaces with higher concentrations of $OH^{\bullet}$ radical ions, MB is removed by a dual phenomenon, namely adsorption/photodegradation, which results in better MB removal from the system. Whileas, in the case of MY, an anionic dye, the accelerated degradation at higher pH can be attributed to higher levels of $OH^{\bullet}$ radical ions created by the photocatalyst, resulting in a more efficient photodegradation process at higher pH.

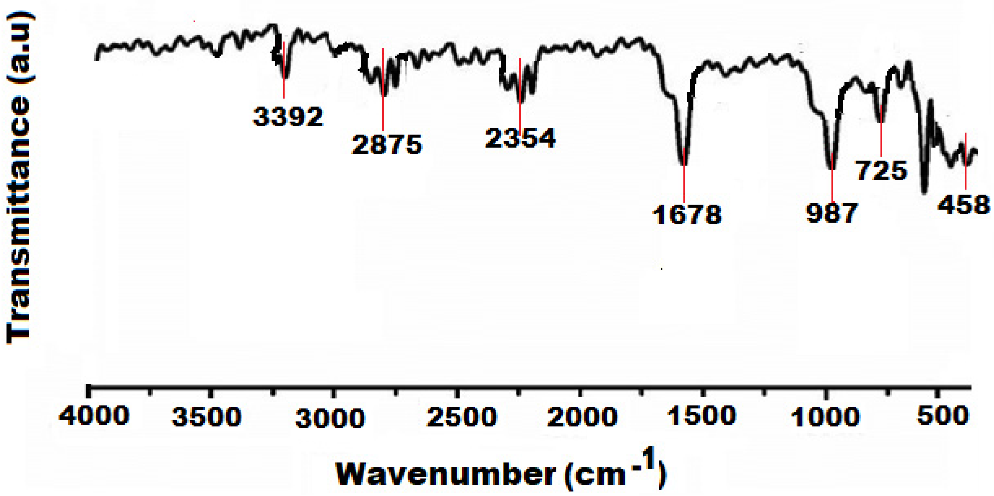

Figure S1. FTIR spectra of biosynthesized Al_2O_3 nanoparticles at 4000–500 cm^{-1} after annealing.

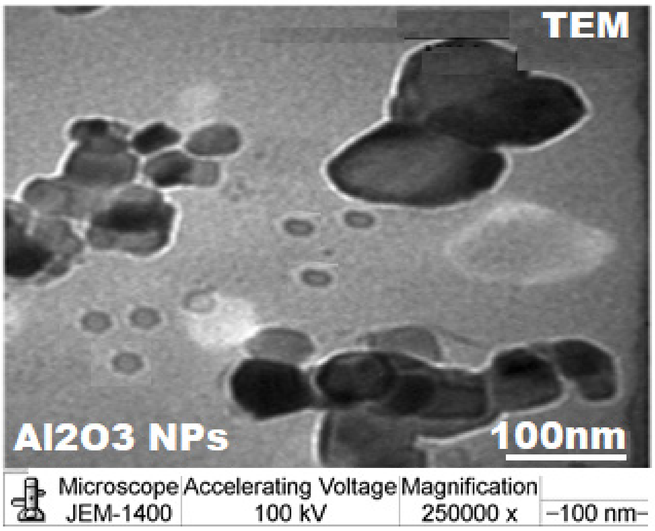

Figure S2. TEM image of Al_2O_3 NPs at ×250,000 magnifications.

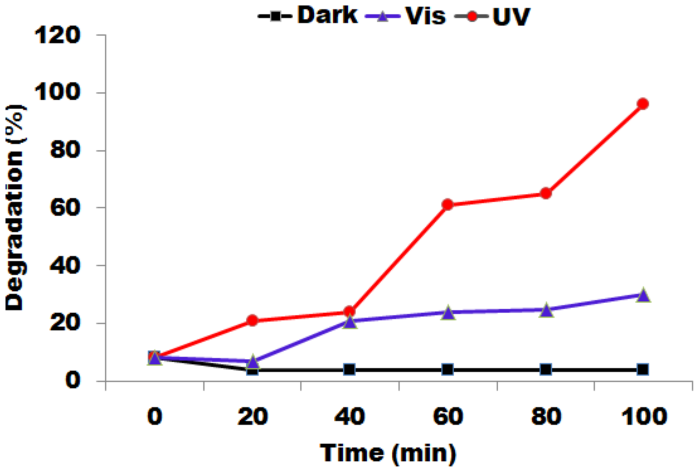

Figure S3. MY photodegradation employing the biosynthesized Al_2O_3 nanoparticles.
